# ABA-Mediated Stomatal Response in Regulating Water Use during the Development of Terminal Drought in Wheat

**DOI:** 10.3389/fpls.2017.01251

**Published:** 2017-07-18

**Authors:** Renu Saradadevi, Jairo A. Palta, Kadambot H. M. Siddique

**Affiliations:** ^1^School of Agriculture and Environment, The University of Western Australia, Perth WA, Australia; ^2^The UWA Institute of Agriculture, The University of Western Australia, Perth WA, Australia; ^3^CSIRO Agriculture and Food, Wembley WA, Australia

**Keywords:** abscisic acid, stomatal conductance, water use efficiency, root hydraulic conductivity, grain yield

## Abstract

End-of-season drought or “terminal drought,” which occurs after flowering, is considered the most significant abiotic stress affecting crop yields. Wheat crop production in Mediterranean-type environments is often exposed to terminal drought due to decreasing rainfall and rapid increases in temperature and evapotranspiration during spring when wheat crops enter the reproductive stage. Under such conditions, every millimeter of extra soil water extracted by the roots benefits grain filling and yield and improves water use efficiency (WUE). When terminal drought develops, soil dries from the top, exposing the top part of the root system to dry soil while the bottom part is in contact with available soil water. Plant roots sense the drying soil and produce signals, which on transmission to shoots trigger stomatal closure to regulate crop water use through transpiration. However, transpiration is linked to crop growth and productivity and limiting transpiration may reduce potential yield. While an early and high degree of stomatal closure affects photosynthesis and hence biomass production, a late and low degree of stomatal closure exhausts available soil water rapidly which results in yield losses through a reduction in post-anthesis water use. The plant hormone abscisic acid (ABA) is considered the major chemical signal involved in stomatal regulation. Wheat genotypes differ in their ability to produce ABA under drought and also in their stomatal sensitivity to ABA. In this viewpoint article we discuss the possibilities of exploiting genotypic differences in ABA response to soil drying in regulating the use of water under terminal drought. Root density distribution in the upper drying layers of the soil profile is identified as a candidate trait that can affect ABA accumulation and subsequent stomatal closure. We also examine whether leaf ABA can be designated as a surrogate characteristic for improved WUE in wheat to sustain grain yield under terminal drought. Ease of collecting leaf samples to quantify ABA compared to extracting xylem sap will facilitate rapid screening of a large number of germplasm for drought tolerance.

## Introduction

Wheat (*Triticum aestivum* L.) is the second most important dietary intake grain after rice (FAO, 2013) and the most internationally traded food crop (Foresight, 2011). Average annual global production reached 713 million metric tons in 2013 (FAO, 2015) and around 65% of the produce is used as food (FAO, 2013). By 2050, wheat production has to double to meet the growing global demand for food (Foresight, 2011). Achieving this target, against rising global temperatures and changing patterns of precipitation, will be challenging. Drought is a major abiotic stress reducing wheat yields in many wheat growing areas of the world. Although drought at all wheat growth stages impair crop performance, drought occurring during flowering and grain-filling (terminal drought) is the most detrimental to grain yield.

Terminal drought often occurs in wheat growing regions with Mediterranean-type climatic conditions. These regions are characterized by wet, cold winters and dry, warm summers and wheat growth is low during winter due to low temperature and radiation (Palta and Watt, 2009). The low transpiration demand due to low temperature, low vapour pressure deficit (VPD) and low variability in winter rainfall reduces the occurrence of water stress during vegetative growth. Low and erratic rainfall, increased temperatures and VPD, and evaporative demand in spring and early summer lead to soil water shortage, which often causes crop water deficit after flowering (Turner and Asseng, 2005). Thus, terminal drought is the most significant stress affecting wheat yield (Saini and Aspinall, 1981), but the degree of grain yield reduction depends on the time and rate of development of the crop water deficit (Kobata et al., 1992; Palta et al., 1994). Grain yield in wheat declined by 50% when terminal drought was induced at flowering (Dias de Oliveira et al., 2013). Under extreme terminal drought conditions, wheat yields can fall below 0.5 t/ha (Asseng et al., 2004). Reduced rainfall predicted during autumn may delay sowing until later in the season and could, therefore, further increase the risk of exposure to terminal drought (Farre and Foster, 2010). The impact of water stress on wheat yield is determined by how it affects the physiological processes and conditions in plants, which varies between wheat genotypes (Kramer, 1980).

In water-limited environments, grain yield is a function of water use, water use efficiency (WUE) and harvest index (Passioura, 1983). Hence, terminal drought can be contested to a considerable extent by breeding new varieties with traits that improve WUE (Turner and Asseng, 2005). WUE describes the biomass accumulated per unit of water consumed, and is often used in different levels and units (Turner, 1986; Tambussi et al., 2007). Reduced water uptake will clearly improve WUE, but reduces yield as per Passioura’s equation described above. For improving yield under water limited environment, identifying traits that favor effective use of available water is considered essential (Blum, 2009). This viewpoint article explores the possibilities of exploiting potential genotypic differences in ABA response to soil drying in regulating the use of water to protect yield under terminal drought.

## Crop Adaptive Strategies to Combat Terminal Drought

Terminal drought occurs when crops enter their reproductive growth stage (Turner and Begg, 1981). Since wheat is a determinate crop (Atwell et al., 1999), adaptation mechanisms such as reductions in leaf area, tiller number and biomass are no longer feasible under terminal drought. Drought escapism, the ability to complete a lifecycle before severe plant water deficit develops (Kramer, 1980), has been used by crop breeders for earliness (Siddique et al., 1989). However, earliness may reduce yield potential in years where rainfall is plentiful (Turner, 1986). Furthermore, under Mediterranean-type climates, drought escapism should be accompanied by low-temperature tolerance (Kramer, 1980). Under prevailing unpredictable rainfall conditions, adaptive measures to tolerate drought either by postponing or enduring dehydration (Turner, 1986) help to sustain physiological activities and minimize yield loss in instances where rainfall is minimal. Osmotic adjustment to tolerate dehydration has no direct influence on grain yield other than modifying the water extraction pattern (Morgan and Condon, 1986; Serraj and Sinclair, 2002). Furthermore, osmotic adjustment helps plants to keep stomatal open under water stress (Blum et al., 1999), which could rapidly exhaust available soil water and be detrimental to grain filling and yield.

Terminal drought affects grain filling (Fischer and Kohn, 1966; Saini and Aspinall, 1981; Rajala et al., 2009), resulting in shriveled grains (Mitchell et al., 2013). The carbohydrate requirement for grain filling is partly met by current assimilates and partly by the translocation of assimilates stored in vegetative parts. Under terminal drought, the major source of carbon for grain filling is stored assimilates in the tillers (Pheloung and Siddique, 1991; Kobata et al., 1992; Blum, 1998) as photosynthesis will be limited by water stress. The proportion of biomass converted to grain yield is determined mainly by the water used after anthesis (Passioura, 1983). Thus, every extra millimeter of water extracted during grain filling can result in yield advantage (Manschadi et al., 2006; Kirkegaard et al., 2007). Therefore, sustaining water uptake during grain filling is critical for improving grain yield under terminal drought. Plant adaptation strategies such as stomatal closure to regulate water loss and/or root properties to slow down rapid depletion of soil moisture use may lead to yield improvement under terminal drought.

## Stomatal Regulation to Control Water Use Under Terminal Drought

More than 90% of water uptake in plants is lost through transpiration (Pei et al., 1998) mainly through diminutive pores in the leaf epidermis called stomata. Leaf transpiration is determined by the leaf-to-air vapor pressure deficit (VPD) and resistance to the movement of water from the leaf to the atmosphere (Farquhar and Sharkey, 1982). Reducing the width of the stomatal opening reduces the ease with which water passes from the plant to the atmosphere (stomatal conductance) and is considered a drought adaptive mechanism (Schmidt, 1983).

## Root-To-Shoot Signaling to Regulate Stomata

Stomatal regulation in response to soil dryness implies communication between the roots in the drying soil and the responding leaves. As roots are in direct contact with the drying soil, it has been postulated that roots generate and transmit signals to the leaves such that the stomata respond (Gollan et al., 1986; Passioura, 1988; Blum and Johnson, 1993). The involvement of root signals in controlling stomata has been confirmed by many studies and a vast pool of data supports a chemical signal, the plant hormone abscisic acid (ABA) (Loveys and Kriedemann, 1974; Zhang et al., 1987; Henson et al., 1989b; Zhang and Davies, 1990a; Munns and Sharp, 1993).

Abscisic acid has been strongly advocated as the chemical signal involved in this root-to-shoot communication process, but it has not been confirmed as the sole signal involved. For instance, Munns and King (1988) showed the presence of a different compound in the xylem sap of wheat plants that reduces stomatal conductance and increases leaf ABA concentration. When excised wheat leaves were fed exogenous solutions without ABA, partial stomatal closure was noticed (Dodd, 2013), probably due to the lack of some signals to keep the stomata fully open, possibly other hormones like cytokinin. In recent years, hormone interactions (Acharya and Assmann, 2009) and interactions between hormones and the environment have attracted much interest. Thus, the involvement of other hormones and chemicals like cytokinin, auxins, ethylene, jasmonic acid, salicylic acid, H_2_O_2_ and ionic substances has been suggested which can act either as positive (presence or increased concentration causes stomatal closure) or negative (absence or decreased concentration reduces stomatal conductance) signals (Schachtman and Goodger, 2008; Acharya and Assmann, 2009; Wilkinson et al., 2012). Esters of ABA, especially glucose esters, can play a significant role as a root signal (Munns and Sharp, 1993; Sauter et al., 2002). An increase in xylem pH (Davies and Zhang, 1991; Sobeih et al., 2004) has also been considered a root signal or an amplifier of root signal which facilitates the redistribution of sequestrated leaf ABA to reach guard cells.

Another study with grafted *Arabidopsis* plants with either ABA-deficient stock or scion points to little importance of ABA as a root signal, but emphasized the importance of leaf ABA in stomatal regulation (Christmann et al., 2007). Supplying water directly to leaves of water-stressed plants reverted stomatal closure indicating that hydraulic signals were also involved in stomatal regulation (Comstock, 2002; Christmann et al., 2007). A drop in root water potential, with a net result of decreased soil water potential and water flux, can be considered the signal generator to regulate stomata (Tardieu et al., 1991). No consensus has been reached regarding the root signal that causes stomatal closure when the soil dries. Whatever it may be, ABA concentration in wheat leaves increases in response to water stress (Wright, 1969) and modulates stomatal conductance (Mittelheuser and Van Steveninck, 1969).

## Root Distribution in Stomatal Regulation

As stomatal closure under water deficits is in response to the signals generated and transmitted from the roots, root characteristics might play an important role in this signal generation process. Wheat plants regulated stomata in response to drying signals from the roots in the top drying layer of the soil profile even though leaf water status was maintained by unlimited water supply from deeper soil layers (Blum and Johnson, 1993; Saradadevi et al., 2015). These findings were substantiated with increased ABA concentration in barley leaves when more seminal roots were distributed in the dry half of the pots (Martin-Vertedor and Dodd, 2011). This proves that root distribution plays an important role in signal generation and subsequent stomatal regulation. Therefore, under terminal drought conditions in Mediterranean-type regions, a greater root distribution in the drying upper soil layers causes ABA to accumulate in leaves which regulates stomata to conserve water for grain filling. This may help the plant as an early signaling mechanism to regulate stomata and conserve water well before a large part of the root zone has been depleted of water.

## ABA Accumulation and Stomatal Regulation

An increased concentration of ABA in leaves associated with reduced stomatal conductance (g_s_) under water deficits has been confirmed in several studies conducted in various species including wheat (Wright, 1969; Loveys and Kriedemann, 1974; Quarrie and Jones, 1977; Quarrie, 1980; Blackman and Davies, 1985; Zhang et al., 1987; Henson et al., 1989b; Davies and Zhang, 1991; Munns and Sharp, 1993). Leaf ABA as the main driver of stomatal regulation was questioned when several studies in species such as maize demonstrated that xylem ABA increases much earlier than leaf ABA and correlates better with g_s_ than leaf ABA (Blackman and Davies, 1985; Zhang and Davies, 1990a; Tardieu et al., 1992). This is because leaf ABA consists of ABA sequestrated into the mesophyll chloroplast which has no effect on stomatal regulation (Dodd et al., 1996). However, this has not been clearly demonstrated in wheat, probably because few studies have measured xylem sap ABA in wheat under drying soil conditions (**Table 1**) as a consequence of the difficulty in obtaining xylem sap (Cramer and Lewis, 1993; Munns et al., 1993). In addition, strong correlation between leaf ABA and g_s_ has been demonstrated in wheat (Henson et al., 1989b; Ali et al., 1998; Saradadevi et al., 2014, 2015), unlike in maize or sunflower (Zhang and Davies, 1990b; Tardieu et al., 1992). This does not suggest that xylem ABA has no role in stomatal regulation in wheat. The limited studies that have extracted xylem sap from wheat seedlings by pressuring the whole plant have demonstrated that xylem sap ABA increases with reduction in soil moisture, and turgid wheat leaves reduce g_s_ when fed the collected sap (Munns and King, 1988; Munns et al., 1993). Wheat leaves fed with exogenous ABA also mimicked the effect of water stress by closing their stomata (Mittelheuser and Van Steveninck, 1969; Quarrie and Jones, 1977), confirming the involvement of xylem ABA in the stomatal regulation of wheat. However, the exogenous ABA concentration required to mimic stomatal response was 100 times that of its endogenous ABA (Munns and King, 1988) indicating that other factors act in conjunction with xylem ABA in stomatal closure, such as the presence of other compounds (Munns et al., 1993) or xylem sap pH (Wilkinson and Davies, 1997; Sobeih et al., 2004). Alternatively, leaf ABA may contribute to ABA that reach guard cells in water stressed plants (Cowan et al., 1982; Bahrun et al., 2002), especially in mature plants since stomatal sensitivity to xylem ABA decreases with aging in wheat (Atkinson et al., 1989). Increased accumulation of leaf ABA in non-pressurized plants compared to pressurized wheat plants under similar moisture stress supports the leaf as the major source for ABA at the reproductive stage (Westgate et al., 1996). Flag leaf ABA increases in response to turgor loss and is the source for ABA to the spike (Morgan and King, 1984). Consequently, at least in wheat plants at the reproductive stage, leaf ABA is significant and correlated with g_s_ (Henson et al., 1989b). Evidence from different species including wheat suggests that stomatal regulation can be considered the net result of an integrative response of both root and leaf ABA (Tardieu and Davies, 1993).

**Table 1:** Examples of previous research conducted in wheat to elucidate the role of ABA under drought.

Sl. no.	Stage of plant	Methodology of drought initiation	Tissue sampled for ABA analysis	Exogenous ABA application	Application method	Concentration of exogenous ABA	Reference
1	Seedling	Wilting excised leaf	Leaves	No	–	–	Wright, 1969
2	Seedling	Withholding water	–	Yes	Injection to leaf sheath	3.8 × 10^-4^ M	Quarrie and Jones, 1977
3	Vegetative reproductive	Withholding water	Leaves	Yes	Soil drenching	10^-6^ M	Du et al., 2013
4	Reproductive	Withholding water	Spikes	Yes	Injection through leaf sheath	10^-4^M	Ji et al., 2011
5	Seedling	No drought treatment	Xylem sap	Yes	Added to nutrient medium	10^-5^ M	Kudoyarova et al., 2011
6	Reproductive	Water stress in field	–	Yes	Foliar sprays	10^-3^ M	Travaglia et al., 2010
7	Reproductive	Water stress in field	–	Yes	Foliar sprays	300 mg L^-1^	Travaglia et al., 2007
8	Seedling	No drought treatment	Sap and roots				Vysotskaya et al., 2003
9	Flag leaf	Withholding water	Flag leaves, floral organs	No			Westgate et al., 1996
10	Stem elongation	No drought treatment	–	Yes	Detached leaf feeding root medium	10^-4^M	Blum and Sinmena, 1995
11	Seedling	No drought treatment	–	Yes	Detached stem feeding	10^-3^M	Dodd and Davies, 1994
12	Seedling	Withholding water	Xylem sap	No	–	–	Munns et al., 1993
13	Reproductive	Withholding water	Spikelets	Yes	Through a wick threaded through peduncles	500 μL	Dembinska et al., 1992
14	Reproductive	Withholding water	Flag leaves	Yes (to lupin)	Excised leaf feeding	10^-4^ to 10^-2^mol m^-3^	Henson et al., 1989a
15	Seedling	No drought treatment	–	Yes	Injection into mid vein of leaf	10^-2^ and 10^-3^ mol m^-3^	Atkinson et al., 1989
16	Reproductive	Withholding water	Leaves Spikes	No	–	–	Morgan and King, 1984
17	Reproductive	Withholding water	Leaves Spikes	Yes	Immersing leaf in ABA solution	10 and 30 mg L^-1^	Morgan, 1980
18	Reproductive	Withholding water	Leaves	No	–	–	Innes et al., 1984
19	Jointing and Booting	Withholding water	Leaves	Yes	Soil drench	10 μM	Du et al., 2013
20	Reproductive	Withholding water	Leaves	No	–	–	Saradadevi et al., 2014
21	Reproductive	Withholding water	Leaves	No	–	–	Saradadevi et al., 2015

## ABA Regulates Root Hydraulic Resistance: A Trait that Limits Water Flux Through Roots

Water flow through plants is governed by the driving forces and resistance imposed by the conduit (Boyer, 1985). Considerable resistance to water flow through the plant is provided by roots (Newman, 1976). Therefore, resistance to water flow (low conductance) within the root prevents absorption and the supply of water to the shoot even though root growth is sufficient to reach available water within the soil. Water absorbed by roots flows across the root radius to reach xylem (radial pathway) and then follows a longitudinal pathway to the shoot through the xylem (axial pathway). Hence, root hydraulic resistance is a combination of resistances offered by both radial and axial pathways, with radial flow being the greatest constraint (Steudle and Peterson, 1998; Bramley et al., 2009). Root structure and anatomy contributes to the hydraulic properties of roots (Bramley et al., 2009). For instance, small xylem vessels impart larger resistance to water flow through the xylem (Richards and Passioura, 1989). Likewise, the predominant radial pathway adopted affects hydraulic conductance. For example, apoplastic flow is driven by the hydrostatic gradient and involves minimal resistance compared with the symplastic pathway (Steudle and Peterson, 1998). In wheat, significant radial water flow occurs symplastically (Bramley et al., 2009), which is facilitated by the membrane-bound protein, aquaporin. Aquaporin activity can potentially be enhanced by interactions with ABA (Hose et al., 2000). A higher concentration of ABA was observed in wheat roots in association with increased root hydraulic conductance following excision of four out of five seminal roots (Vysotskaya et al., 2003, 2004). This hike in root ABA and subsequent enhancement of root hydraulic conductivity to meet increased transpiration demand is due to the redistribution of ABA from leaf to root (Kudoyarova et al., 2011). Thus, leaf ABA is involved in regulating root hydraulic conductivity, in addition to its role in regulating stomata.

## ABA Dynamics in Plants

Abscisic acid is synthesized in apical root cells and also in mesophyll cells in the leaves (Hartung et al., 2002). Plant roots absorb ABA and its conjugates (ABA-glucose ester) from the soil solution (Hartung et al., 2002). Root cells synthesize ABA when their water status is reduced by 50% or more (Hartung et al., 2002). ABA in the root tissues takes both apoplastic and symplastic pathways to reach xylem (Hartung et al., 2002). Xylem ABA acts as an early signal that initiates stomatal regulation (Zhang and Davies, 1990a). As water deficit increases, ABA biosynthesis in leaves is triggered by a reduced leaf water potential or turgor (Westgate et al., 1996). ABA concentration increases in all leaf tissues including guard cells (Harris et al., 1988). Leaf-synthesized ABA is loaded into the phloem and transported to the roots (Slovik et al., 1995; Kudoyarova et al., 2011) either to enter the xylem (Liang et al., 1997) or to be deposited in root tissues (Hartung et al., 2002). During transportation from root to leaves, stem parenchyma cells also contribute to xylem ABA under conditions of high concentration and pH gradient (Sauter and Hartung, 2002). ABA being a weak acid (Hartung and Slovik, 1991), ABA reaching the leaf lamina through xylem gets sequestrated into alkaline compartments of leaf tissues (Cowan et al., 1982; Slovik et al., 1995) depending on the pH gradient between the tissue and xylem (Wilkinson and Davies, 1997). With higher xylem sap pH, ABA sequestration to leaf tissue is reduced or the redistribution of leaf tissue ABA to reach guard cells is favored (Cowan et al., 1982; Popova et al., 2000). In addition, guard cells can synthesize ABA (Bauer et al., 2013).

**FIGURE 1:**
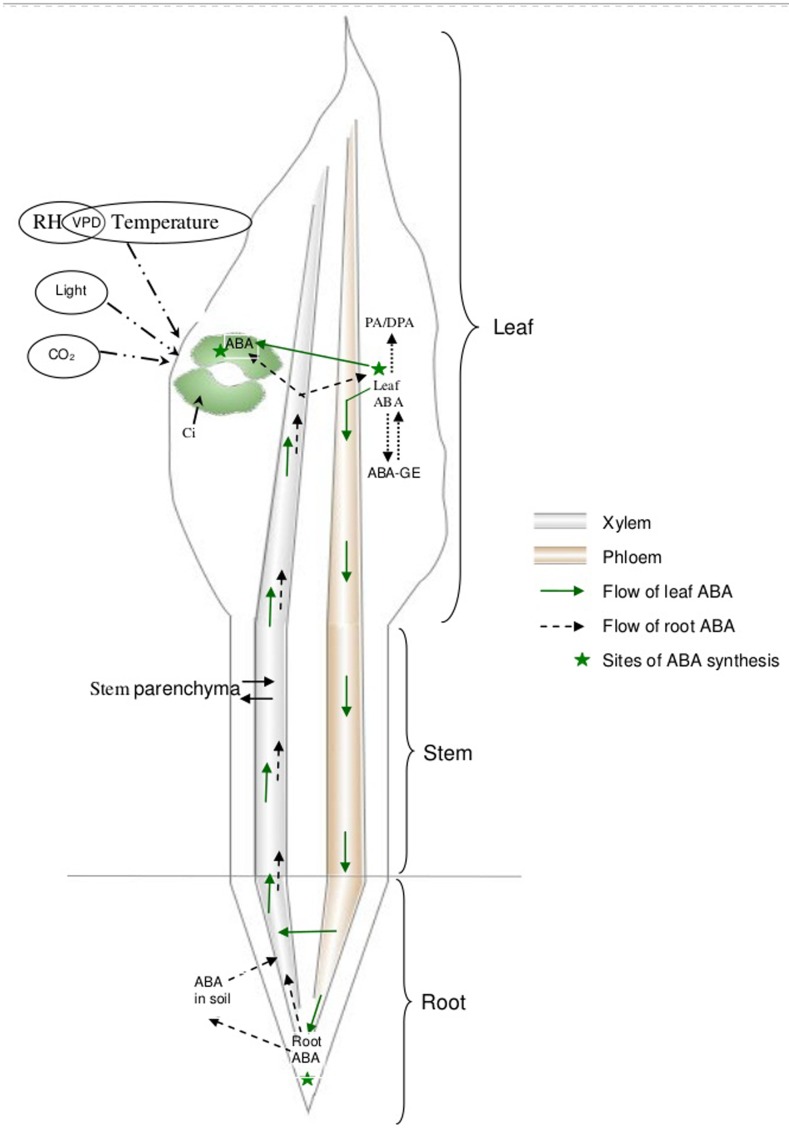
Schematic representation of the kinetics of ABA through the plant.

Abscisic acid also gets degraded to form phaseic acid (PA), which may be further metabolized to dihydrophaseic acid (DPA) (Harrison and Walton, 1975; Creelman and Zeevaart, 1984). Alternatively, ABA conjugates with glucose to form ABA-glucose ester (ABA-GE) which is not active in stomatal regulation (Zeevaart and Creelman, 1988). Esters of ABA are present in the xylem sap of several species (Jeschke et al., 1997; Hansen and Dörffling, 1999; Sauter et al., 2002) and are believed to be involved in root-to-shoot signaling. In wheat, the high-molecular weight compound with anti-transpiration properties in the xylem sap of water-stressed plants is possibly a glucose ester of ABA (Munns and King, 1988; Munns et al., 1993). ABA-GE is capable of releasing free ABA upon hydrolysis by β-glucosidases (Dietz et al., 2000; Lee et al., 2006; Schroeder and Nambara, 2006; Xu et al., 2012). Thus, bulk leaf ABA is the net result of ABA transport through the xylem, its biosynthesis in leaves, degradation and conjugation (**Figure 1**). To understand the mode-of-action of ABA, ABA biosynthesis, distribution, and degradation, it is critical to first establish reliable tissue sampling techniques to quantify ABA.

## Tissue Sampling for ABA Analysis

A strong correlation exists with ABA and g_s_, but the relationship varies with species and the tissue sampled. The main reason for this discrepancy is that the ABA concentration in leaf tissue or xylem sap does not relate to those reaching guard cells, which acts on stomata (Munns and Sharp, 1993). The most accurate option is to measure ABA nearest to the site of action, which is the guard cells, but this is possible only in some species like *Commelina* where the epidermis can be stripped easily (Blackman and Davies, 1983) and guard cells can be isolated by killing all other epidermal cells by a low pH treatment (MacRobbie, 1980). Due to this limitation in isolating the epidermis in some species and the laborious process involved in collecting sufficient epidermal tissue for analysis, xylem sap is considered the best option to explain stomatal behavior (Dodd et al., 1996).

Xylem sap sampling is not easy in cereals as it is difficult to collect a sufficient volume of xylem sap to quantify ABA (Dodd and Davies, 1996), especially in wheat and barley (Cramer and Lewis, 1993; Munns et al., 1993). Hence, most studies where xylem sap ABA is measured have been conducted on maize due to the ease of extraction of a large volume of sap (Dodd and Davies, 1996; **Table 2**). Besides, much attention is needed to get a representative sample as ABA concentration varies with the volume of collected sap (Borel and Simonneau, 2002), the method of collection (Quarrie and Lister, 1983), the point of collection (Netting et al., 2012) and the time of collection (Schurr et al., 1992; Tardieu and Davies, 1992).

**Table 2:** Summary of the literature showing methodology used to collect xylem sap from different species.

Sl. no.	Species	Stage of plant	Drought	Pot/field conditions	Sap collection technique	Remarks	Reference


1	Wheat	Flag leaf stage	Yes	Lysimeter	Root exudation	–	Ali et al., 1998


2	Wheat, barley	Seedling	Yes	Pots	Whole pot in pressure chamber	Difficult from wheat	Munns et al., 1993


3	Wheat	Seedling	Yes	Pots	Whole pot in pressure chamber	–	Munns and King, 1988


4	Wheat, barley	Seedling	Yes	Pots	Whole pot in pressure chamber	–	Munns, 1992


5	Wheat, maize	1-month old plants	No	Hydroponics	Wheat: pressurizing shoots Maize: root exudation	Wheat did not yield any root exudates	Cramer and Lewis, 1993


6	Durum wheat	Seedlings	No (roots severed)	Hydroponics	Root exudation	Cut stump reunited with stem by tubing	Vysotskaya et al., 2003


7	Barley	7 days after transplanting	Yes	Pot	Pressurizing whole plant in pressure chamber	No sap extraction possible under root pressure	Martin-Vertedor and Dodd, 2011


8	Barley	3-weeks-old plants	Yes	Pots	Root exudation	Droplets for pH measurement	Bacon et al., 1998


9	Maize	Seedlings	Yes	Pots	Whole pot in pressure chamber	–	Liang et al., 1997


10	Maize	Flowering	No	Pots	Stem bleeding, Root exudation, aspiration	Bleeding sap often unobtainable	Canny and McCully, 1988


11	Maize	Seedlings	Yes	Pots	Pressurizing cut stem	–	Wilkinson et al., 2007


12	Maize	4 weeks after sowing	Yes	Pots	Root exudation	Maize sap fed to wheat leaves	Zhang and Davies, 1991


13	Maize	Silking	Yes	Field	Over pressurizing leaves (0.5 MPa)	–	Tardieu and Davies, 1992


14	Maize, sunflower	5–6 weeks after sowing	Yes	Pots	Centrifugation^∗^ pressurizing whole root system (sunflower); root exudate under root pressure (maize)	Sunflower plants did not yield enough exudates under root pressure	Zhang and Davies, 1990b


15	Maize	Seedling	Yes	Pots	Root exudation	–	Zhang and Davies, 1990a


16	Maize	Silking	Yes	Field	From leaf by over pressurizing (0.5 MPa) after leaf water potential measurement	–	Tardieu et al., 1992


17	Maize	28 days after emergence	Yes	Lysimeter	Root pressure	–	Bahrun et al., 2002


18	Sunflower	Seedling	^∗∗^PRD	Pots	Whole pot in pressure chamber	–	Dodd et al., 2008a


19	Sunflower	Seedling	PRD	Pots	Whole pot in pressure chamber	–	Dodd et al., 2008b


20	Sunflower	Flowering	Yes	Field	From leaf by over pressurizing (0.3 MPa) after leaf water potential measurement	–	Tardieu et al., 1996


21	Grape	Mature	Yes	Field	From leaf by over pressurizing (0.1 MPa)	–	Speirs et al., 2013


22	Grape	4-months-old plants	PRD	Split roots (two pots)	Whole root system removed from pot and inserted into specially designed pressure chamber	–	Li et al., 2010


23	Tomato	5–6 weeks	PRD	Split roots (two pots)	From leaf petiole in pressure chamber; From petiole and root stubs when whole plant was pressurized	–	Netting et al., 2012


24	Tomato	6 weeks after germination	PRD	Split roots (two pots)	From leaf by over pressurizing (0.4 MPa for 60–120 s) after leaf water potential measurement	–	Dodd et al., 2006; Dodd, 2007


25	Tomato	6 weeks after germination	PRD	Split root	From leaf by over pressurizing (0.2 to 0.4 MPa) after leaf water potential measurement	–	Sobeih et al., 2004


26	Cotton	Fruiting	No	Field	From leaf by over pressurizing	–	Hartung et al., 1988


27	Nicotiana	Flowering	Yes	Pots	From leaf by over pressurizing (0.5 MPa) after leaf water potential measurement	–	Borel and Simonneau, 2002

## Xylem Sap Sampling Techniques

A commonly used method to collect exuded root xylem sap is from detached root stumps (Zhang and Davies, 1990a; Vysotskaya et al., 2003). Removing the aerial part will cease transpiration, which increases root pressure, thus causing exudation. However, little exudate was collected from wheat grown under hydroponics, even without water stress (Cramer and Lewis, 1993). Root exudation did not yield any sap from barley plants (Martin-Vertedor and Dodd, 2011). Nevertheless, Ali et al. (1998) collected xylem sap from water-stressed wheat plants grown in lysimeters through root exudation. The drawback in exudate collection is that the flux of sap exudation will be much lower due to lack of transpiration pull (Schurr, 1998), which alters the ABA concentration in the sap (Else et al., 1994; Goodger et al., 2005). Information about fluxes and ABA concentration is needed (Schurr, 1998) to account for the changes in stomatal opening. It is ideal to collect the sap when the flow rate is similar to the transpiring rate of an intact plant (Else et al., 1995), but negative pressure in the xylem of transpiring plants makes collection difficult (Schurr, 1998).

The pressurization technique allows sap collection to occur at a similar rate of flux as the intact transpiring plant (Munns et al., 1993), but the ABA concentration may vary due to wounding (Else et al., 1994) and the interrupted flow of signals and ions from phloem to xylem (Schurr, 1998). Pressurization can be applied to extract sap from other plant parts like leaves, but the volume of extraction without water contamination from internal compartments is limited (Dodd, 2007). Some studies have collected xylem sap from wheat by pressurizing the whole root system in a pressure chamber (**Table 2**). The lack of pressure chambers (Dodd, 2007) suitable for large flowering stage plants limits its applicability to seedlings.

The application of external forces such as a vacuum is another method of collecting xylem sap. As the xylem fluid is under less axial resistance compared to the fluid in surrounding tissues, application of a slight force will separate xylem sap increasing the risk of contamination with other fluids. Applying negative pressure through a vacuum stimulates conditions similar to intact transpiring plants (Freundl et al., 1998). However, few experiments have used this technique to extract xylem sap (**Table 2**).

Controversy exists regarding which xylem sap sampling procedures best represents ABA concentration in the xylem sap of a transpiring plant. Since the main site of action is the leaves and they are easy to access and abundant (Dodd et al., 1996), and because leaf ABA and g_s_ in wheat are correlated, leaves tissue are the most sampled tissue in ABA studies in wheat (**Tables 1**, **2**).

## Genotypic Variation in ABA Accumulation and Stomatal Sensitivity to ABA

Significant genotypic variation in the accumulation of ABA in wheat leaves under water stress has been demonstrated in most of the studies conducted in wheat (Quarrie and Jones, 1977; Henson and Quarrie, 1981; Quarrie, 1981; Ji et al., 2011; Du et al., 2013). Wheat genotypes that accumulate less ABA in their leaves have been associated to drought resistance and those accumulating more ABA have been considered sensitive to drought (Quarrie, 1981; Ji et al., 2011). On the contrary, high leaf ABA accumulating wheat lines demonstrated better WUE for grain yield than low ABA lines (Innes et al., 1984). Genotypic variation in ABA accumulation associated with drought tolerance in pearl millet is more pronounced under well-watered conditions such that genotypes accumulating more ABA under well-watered conditions showed drought tolerance (Kholova et al., 2010). Similarly, in a split-root study, wheat genotype Drysdale, which yielded more than the drought-tolerant line IGW-3262, had higher leaf ABA content under well-watered conditions (Saradadevi et al., 2014, 2015). These contrasting evidences suggest that the ABA-associated drought tolerance is not just through the effect of ABA on stomatal conductance, but other processes that may be affected by ABA (Quarrie and Henson, 1981), such as pollen sterility, translocation of pre-anthesis stored carbohydrates to grain and root hydraulic conductivity.

Wheat genotypes also differ in their stomatal sensitivity to leaf ABA content, as demonstrated by Blum and Sinmena (1995) through a transpiration bioassay. Thus, differences in stomatal sensitivity to ABA concentration could either counteract or uphold the effect of differences in ABA accumulation among genotypes. Despite clear evidence for genotypic variation in ABA accumulation and sensitivity, information on its impact on stomatal regulation and water use to sustain grain yield is very limited. The potential role of ABA in improving WUE is highlighted by the differential performance of four transgenic wheat lines with and without expression of ABA-responsive barley genes (Sivamani et al., 2000). Recently, we found consistent variation between two genotypes (Drysdale and IGW-3262), in their relationship between leaf ABA, stomatal conductance, water use and yield (Saradadevi et al., 2014, 2015, 2017). As genotypic variation for the capacity to accumulate ABA and ABA sensitivity is highly heritable and homogeneity can be achieved within a few generations (Quarrie, 1981; Quarrie and Lister, 1983), it is important to explore the causes of these genotypic differences.

## Factors Affecting Genotypic Differences in ABA

In the above mentioned studies demonstrating genotypic variation in ABA accumulation, the relationship between leaf ABA and stomatal conductance was stronger in Drysdale, but weaker in IGW-3262. The observed differences between genotypes in the root density distribution in the upper drying soil layer and ABA catabolism was considered as reasons for the differences in leaf ABA and stomatal behavior between these genotypes (Saradadevi et al., 2015). Under well-watered conditions, leaf ABA concentration in IGW-3262 was found to be fluctuating with the relative humidity, but not in Drysdale. Hence genotypic differences in their sensitivity to environmental factors like relative humidity or VPD (Kholova et al., 2010) can also affect ABA accumulation and degradation.

As ABA is not the sole phytohormone involved in stomatal regulation, but several other phytohormones and/or compounds are also involved, the differences among genotypes in the accumulation and sensitivity to ABA might be due to difference among genotypes in the synthesis and degradation of other phytohormones involved in the process. In addition, several factors determine ABA accumulation and the stomatal response to a given concentration of ABA: including the water status of plants, leaf water potential (Quarrie, 1980), leaf turgor (Morgan and King, 1984), soil water status, pH (Slovik et al., 1995; Hartung et al., 2002), soil compaction (Tardieu et al., 1992), environmental factors such as temperature (Wright, 1969; Dodd and Davies, 1994), relative humidity, light or time of day, changes in water flux through the xylem (Slovik et al., 1995) and previous exposure to ABA flux (Atkinson et al., 1989). In addition, the ABA concentration reaching guard cells at any given time can vary due to sequestration, remobilization and degradation and/or conjugation; these mechanisms are not yet fully understood. Furthermore, ABA is mobile within the plant moving up and down the plant; from roots to leaves through xylem and from leaves to roots through phloem. ABA from leaves is also exported to spikes in wheat. Regulation of this movement and its physiological implications is not yet clear (Seo and Koshiba, 2011). Thus, ABA accumulation in response to drought can be confounded effect of two or more of the above factors. In addition, stomata are controlled by several feedback loops (Raschke, 1975) involving external and internal factors such as light, VPD, intercellular CO_2_ concentration, leaf turgor and soil water status. Finally, the role of ABA is not limited to stomatal regulation, but is involved in many physiological functions from seed germination, growth (Milborrow, 1967), tiller production (Quarrie and Jones, 1977), root hydraulic conductivity (Davies et al., 1982), cell wall rigidity (Davies et al., 1982), pollen sterility, and the determination of yield (Travaglia et al., 2007). The involvement of ABA in stomatal regulation and drought adaptation is equivocal, but the above suggested intricacies have limited the understanding of the complex mechanism of ABA-mediated plant responses under water stress. Recent advances in ABA-related research explores biochemical, molecular and genetic aspects of ABA signaling like ABA biosynthesis, ABA receptors, their structures, mechanics of binding ABA to receptors, ABA transporters, gene expressions and transcription factors [reviewed by Cutler et al. (2010), Hubbard et al. (2010), Klingler et al. (2010), Weiner et al. (2010) and Sah et al. (2016)]. Rapid advances in genomic aspects of ABA widens the gap between genomic and physiological information available on ABA in relation to drought adaptation and crop improvement (Blum, 2015). Hence attention is required to explore physiological significance of ABA in combating drought and maintaining grain yield.

## The Effect of Stomatal Regulation on Yield

The main focus of any wheat breeding program is grain yield improvement. Unfortunately, many favorable plant responses to moisture stress have negative effects on grain yield (Schmidt, 1983; Turner, 1986). For instance, Blum and Johnson (1993) showed a negative impact on grain yield of wheat cultivars with reduced stomatal conductance in response to the dry top soil. This may be due to reduced photosynthesis due to stomatal closure or the adverse effect of ABA on pollen sterility showed in other studies (Morgan and King, 1984).

## Stomatal Conductance and Photosynthesis

As stomata serve as a portal through which water exits the leaf and CO_2_ diffuses into leaf tissue for photosynthesis, stomatal regulation to limit water use during water deficit is at the expense of CO_2_ diffusion into leaf tissue which subsequently reduces photosynthesis (Chaves et al., 2009). In addition to the reduced CO_2_ influx, soil water deficits reduce mesophyll conductance of CO_2_ limiting photosynthesis (Flexas et al., 2004). As the relative water content decreases due to soil water deficit, decreased ATP synthesis and consequent RuBP synthesis causes metabolic limitation of photosynthesis (Lawlor, 2002; Lawlor and Cornic, 2002). Interestingly, the rate of reduction of CO_2_ assimilation is comparatively less than the reduction in transpiration (Holaday et al., 1992). In well-adapted plants, the stomatal role in controlling photosynthesis is not more than 20% of the total photosynthetic inhibition (Jones, 1998). So the positive effect of reduced transpiration may outweigh the negative effect of decreased photosynthesis under terminal drought conditions. A low canopy conductance to facilitate water availability for uptake during the reproductive stage is proposed as an important trait to maintain grain yield in chickpea under terminal drought conditions (Guóth et al., 2010).

## Impact of ABA on Yield

Reduction in grain set due to drought has been associated with increased ABA concentrations in leaves and spikes (Morgan and King, 1984). Yield reduction in response to exogenous ABA application supports the negative impact of ABA on grain set and yield (Morgan, 1980). A reduction in the number of grains per ear in genotypes selected for high leaf ABA levels, even under well-watered conditions, suggests that leaf ABA negatively influences wheat pollination (Innes et al., 1984). Therefore, grain yield reduction is observed when the water deficit coincides with pollen mother cell meiosis and not during later developmental stage (Saini and Aspinall, 1981). Furthermore, ABA-associated pollen sterility is more pronounced in drought-sensitive wheat genotypes compared to drought-tolerant ones (Ji et al., 2011). In a split-root study, where the dry half of the root system contributed to increased leaf ABA while the other half supplied water to maintain leaf water potential, no yield reduction was noticed (Dembinska et al., 1992). This suggests that endogenous ABA is not the sole factor affecting grain set under drought.

Conversely, ABA reportedly has a positive influence on grain yield by affecting the redistribution of carbohydrates from the shoot into wheat grain (Travaglia et al., 2007). Increased grain yield by foliar application of ABA in a wheat field was confirmed by Travaglia et al. (2010). This disagrees with the findings of King and Patrick (1982), where no such involvement of ABA in assimilate transport to grain was observed. No grain yield benefit resulted from drenching soil with exogenous ABA (Du et al., 2013). The lack of consensus among researchers in relation to a positive, negative or neutral influence of ABA on grain yield suggests that the timing at which the water stress occurs is important. Pre-anthesis water stress, particularly during spike development and pollen meiosis, reduced grain number while post-anthesis water stress reduced grain size (Dolferus et al., 2011). This is because high ABA levels during the early reproductive stage affect grain set and reduces grain number while during post-anthesis stages, it promotes grain filling by redistributing reserved carbohydrates to the grain (Liu et al., 2005). In a recent study, when the soil water was exhausted rapidly after anthesis, the wheat cultivar Drysdale maintained a higher grain yield with higher harvest index and grain weight compared to the advanced drought-tolerant line IGW-3262 (Saradadevi et al., 2015). This was mainly because the cultivar Drysdale was more efficient at translocation of assimilate to grain (Saradadevi et al., 2015). Based on the above studies, it appears that ABA negatively affects grain set, but has a positive effect on grain filling by facilitating assimilate partitioning to grain.

## Conclusion

Stomatal regulation is an important mechanism that controls water use and maintain grain yield under terminal drought, a abiotic major stress affecting wheat grain yield. The role of ABA in regulating g_s_ under water deficit has been explored extensively in crops such as maize, tomato and sunflower, with limited studies in wheat and no clear consensus on the mechanisms of ABA-mediated stomatal regulation. With contrasting evidence on the relationship between leaf and xylem ABA and stomatal conductance in species like maize and wheat, research should focus on the factors affecting these specific differences and the possibility of exploiting genotypic variation in ABA accumulation as a surrogate characteristic for improving effective water use in wheat to sustain grain yield under terminal drought. Ease of collecting leaf samples to quantify ABA compared to extracting xylem sap will facilitate the rapid screening of a large number of germplasm for drought tolerance.

## Author Contributions

RS has performed data acquisition, analysis and interpretation of the data and drafting of the manuscript. RS is the corresponding author of this manuscript. KS and JP conceived the idea, assisted in data interpretation, and critically reviewed and edited the manuscript.

## Conflict of Interest Statement

The authors declare that the research was conducted in the absence of any commercial or financial relationships that could be construed as a potential conflict of interest.
